# Survival in oral and pharyngeal cancers is catching up with laryngeal cancer in the NORDIC countries through a half century

**DOI:** 10.1002/cam4.6867

**Published:** 2024-01-02

**Authors:** Frantisek Zitricky, Anni I. Koskinen, Otto Hemminki, Asta Försti, Akseli Hemminki, Kari Hemminki

**Affiliations:** ^1^ Faculty of Medicine and Biomedical Center in Pilsen Charles University in Prague Pilsen Czech Republic; ^2^ Department of Otorhinolaryngology‐ Head and Neck Surgery Helsinki University Hospital and University of Helsinki Helsinki Finland; ^3^ Department of Urology Helsinki University Hospital and University of Helsinki Helsinki Finland; ^4^ Cancer Gene Therapy Group, Translational Immunology Research Program University of Helsinki Helsinki Finland; ^5^ Hopp Children's Cancer Center (KiTZ) Heidelberg Germany; ^6^ Division of Pediatric Neurooncology, German Cancer Research Center (DKFZ) German Cancer Consortium (DKTK) Heidelberg Germany; ^7^ Comprehensive Cancer Center Helsinki University Hospital Helsinki Finland; ^8^ Division of Cancer Epidemiology, German Cancer Research Centre (DKFZ) Heidelberg Germany

**Keywords:** conditional survival, human papilloma virus, oral cancer, pharyngeal cancer

## Abstract

**Background:**

Cancers of the head and neck (HN) are heterogeneous tumors with incidence rates varying globally. In Northern Europe oral and oropharyngeal cancers are the most common individual types. Survival for HN varies by individual tumor type but for most of them survival trends are not well known over extended periods of time.

**Methods:**

Data for a retrospective survival study were obtained for Danish, Finnish, Norwegian, and Swedish patients from the NORDCAN database from 1971 to 2020. Relative 1‐ and 5‐year survival rates and 5/1‐year conditional survival for years 2–5 were calculated.

**Results:**

Both 1‐ and 5‐year survival improved for all HN cancers but only marginally for laryngeal cancer. For the other cancers a 50‐year increase in 5‐year survival was about 30% units for nasopharyngeal and oropharyngeal cancers, 20% units for oral cancer and somewhat less for hypopharyngeal cancer.

**Conclusions:**

5‐year survival reached about 65% for all HN cancers, except for hypopharyngeal cancer (30%). Human papilloma virus infection is becoming a dominant risk factor for the rapidly increasing oropharyngeal cancer, the prevention of which needs to emphasize oral sex as a route of infection.

## 1 INTRODUCTION

Cancers of the head and neck (HN) in anatomic sequence include, oral (oral cavity), nasopharyngeal, oropharyngeal, hypopharyngeal, and laryngeal cancers of the upper aerodigestive tract.^1^ These cancers are predominantly squamous cell carcinomas and their incidence is generally higher in men compared to women for which the common risks factors of tobacco smoking, alcohol intake and their interactions are at least a partial explanation.^2^, ^3^ The relative risk of these cancers in active smoker is of the order of 10–15, but the risk is thought be lower (less than 2) for nasopharyngeal cancer.^2^, ^4^ While the overall risk of smoking may be somewhat lower for these cancers than it is for lung cancer, the positive influence of quitting smoking appears to be faster than that for lung cancer, and after 10 years of quitting the excess risk may have disappeared.^2^ The main risk factor for oropharyngeal cancers in industrialized countries is infection with human papillomavirus (HPV), in addition to tobacco and alcohol.^2^, ^3^, ^5^, ^6^ The increasing incidence and male prominence in oropharyngeal cancer have been attributed to an increasing burden of HPV infections through oral sex^7^, ^8^, ^9^, ^10^; it is assumed that over 90% of oral HPV infections are sexually transmitted.^11^, ^12^ HPV‐positivity varied in over 2000 European oropharyngeal cancer patients assessed from publications between 2014 and 2018 from 18% to 65%.^13^ The highest figures were for Denmark and Sweden; Finland was at 50%.

In Denmark, where the prevalence of the above risk factors has been high, the incidence in HN cancers in 1980–2014 has been highest for laryngeal and oral cancers (both 8000 cases), followed by oropharyngeal (6000), hypopharyngeal (2000), and nasopharyngeal (1000) cancers.^9^ In a European study, the ranking was approximately the same but laryngeal cancers were relatively more common.^14^ The main incidence change in HN cancers has been in the vast increase in oropharyngeal cancer which started in Sweden in the 1970s and in Finland 10 years later with male prominence in both countries.^15^ HPV etiology has also clinical implications as HPV‐positive oropharyngeal cancers are more responsive to treatment than HPV‐negative cancers and thus have a more favorable prognosis.^3^, ^9^, ^16^, ^17^, ^18^ Smoking may not worsen survival in HPV‐positive oropharyngeal cancer but may increase the risk of tumor recurrence.^19^, ^20^ Viral etiology is known also for nasopharyngeal cancer which shows internationally endemic clustering in Southern Asia and Northern Africa and is often associated with Epstein–Barr virus positivity; the mortality difference between the highest (Malaysia) and lowest global mortality (Finland) is 50‐fold.^3^, ^21^

We report here historical and up‐to‐date survival data for the selected HN cancers from Denmark (DK), Finland (FI), Norway (NO), and Sweden (SE) over a half century. These countries have long historical and cultural ties and they have organized medical care accessible to the whole population practically free‐of‐charge; yet investment in medical care infrastructure has depended on country‐specific economic resources (www.macrotrends.net). As to the risk factor of HN cancers, the population prevalence of smokers has varied between the countries with SE men emerging as non‐smoking champions towards year 2000 having switched to smokeless tobacco “snus” (www.pnlee.co. uk/ISS.htm).^22^ Alcohol consumption was historically modest in the Nordic countries but after 1970 it increased mostly in DK and FI.^23^ Probably also sexual habits differed between the countries as judged from the vastly higher incidence rates for cervical cancer in DK women in the 1960s and 1970s.^24^

## 2 MATERIALS AND METHODS

The data were obtained from the NORDCAN database 2.0.^25^, ^26^ The database was accessed at the International Agency for Cancer (IARC) website (https://nordcan.iarc.fr/en),^27^ and the available tools were used to extract data on incidence, mortality and 1‐year and 5‐year survival. NORDCAN uses International Classification of Diseases (ICD) version 10 codes. For oral cavity these were C00.3‐C00.5 (upper and lower lip, inner parts), C02‐C04 (other and unspecified parts of tongue, gum, floor of mouth), C05.0 (hard palate), C05.8‐C05.9 (overlapping and unspecified palate) and C06 (other and unspecified parts of mouth). For nasopharynx the code was C11, for oropharynx they were C01 (base of tongue), C05.1‐C05.2 (soft palate, uvula), C09 (unspecified palate), C10.0 (vallecular), C10.2‐C10.9 (lateral, posterior, overlapping and unspecified oropharynx and branchial cleft), C14.0 (unspecified pharynx) and C14.2‐C14.8 (Waldeyer ring, overlapping parts) and for hypopharynx they were C12‐C13 (piriform sinus and hypopharynx). The code for laryngeal cancer was C10.1 (anterior surface of epiglottis) and C32 (larynx).

Using the NORDCAN, we extracted data on 1‐ and 5‐year relative survival, and the follow‐up was extended until death, emigration or loss of follow‐up or to the end of 2020. Survival data for relative survival were available from 1971 onwards and the analysis was based on the cohort survival method for the first nine 5‐year periods, and a hybrid analysis combining period and cohort survival in the last period 2016–2020, as detailed.^25^ Age‐standardized relative survival was estimated using the Pohar Perme estimator.^28^ Age‐standardization was performed by weighting individual observations using external weights as defined at the IARC website. Age groups 0–89 years were considered. The national life tables were used to calculate the expected survival. The detailed methods are described elsewhere.^29^

Comparisons with the US Surveillance, Epidemiology and End Results (SEER) data for years 2012–18 on Non‐Hispanic whites was done through (https://seer.cancer.gov/statistics‐network/explorer/application.html?site=1&data_type=1&graph_type=2&compareBy=sex&chk_sex_3=3&chk_sex_2=2&rate_type=2&race=1&age_range=1&hdn_stage=101&advopt_precision=1&advopt_show_ci=on&hdn_view=0&advopt_display=2#graphArea).

## 3 RESULTS

### 3.1 Patient population

The case numbers and incidence in HN cancers in the NORDCAN database in 2011 to 2020 are shown in Table 1; all these cases were included in survival analysis. In each country male rates were higher than the female ones, for oral and nasopharyngeal cancer about two‐fold higher, for oropharyngeal cancer about three‐fold higher and for hypopharyngeal and laryngeal cancers four‐fold or more. For men, oropharyngeal cancer was the most common HN cancer in countries other than FI where oral cancer was the most common. Oral cancer was the most common female HN cancer. All of these cancers had the highest incidence in DK men and women, and for hypopharyngeal cancer the difference was the largest. The median ages for SE men/women were 67/73 years for oral, 62/63 years for oropharyngeal, 60/65 years for nasopharyngeal, 68/70 years for hypopharyngeal, and 68/67 years for laryngeal cancer; no large differences were noted between the countries.

**TABLE 1:** Age‐standardized incidence rates (world) per 100,000 and case numbers for specific cancer sites during 2011–2020 period in the Nordic countries.

	Denmark		Finland		Norway		Sweden	
Cancer site	Males	Females	Males	Females	Males	Females	Males	Females
Oral cavity	4.4 2257	2.3 1346	3.4 1722	2.2 1431	2.6 1156	1.7 898	2.3 2161	1.8 1973
Nasopharynx	0.38 159	0.17 74	0.25 108	0.10 50	0.27 104	0.17 55	0.30 216	0.16 110
Oropharynx	6.2 3021	2.2 1106	2.7 1307	0.99 515	3.7 1541	1.2 516	3.5 2977	1.4 1148
Hypopharynx	1.6 856	0.33 187	0.55 309	0.09 55	0.45 212	0.11 55	0.44 456	0.12 128
Larynx	3.6 1996	0.85 481	2.0 1095	0.25 162	1.9 940	0.42 194	1.4 1457	0.32 316

### 3.2 Survival trends

In Table S1, 5‐year survival is recorded for each cancer in 5‐year periods for the common cancers of the oral cavity, oropharynx and larynx, and in 10‐year periods for the rare cancers. We use the final period of these results to display the best and the worst country (for men and women) as text figures to highlight the contrasts, and the intermediary countries are shown as supplementary figures.

**FIGURE 1:**
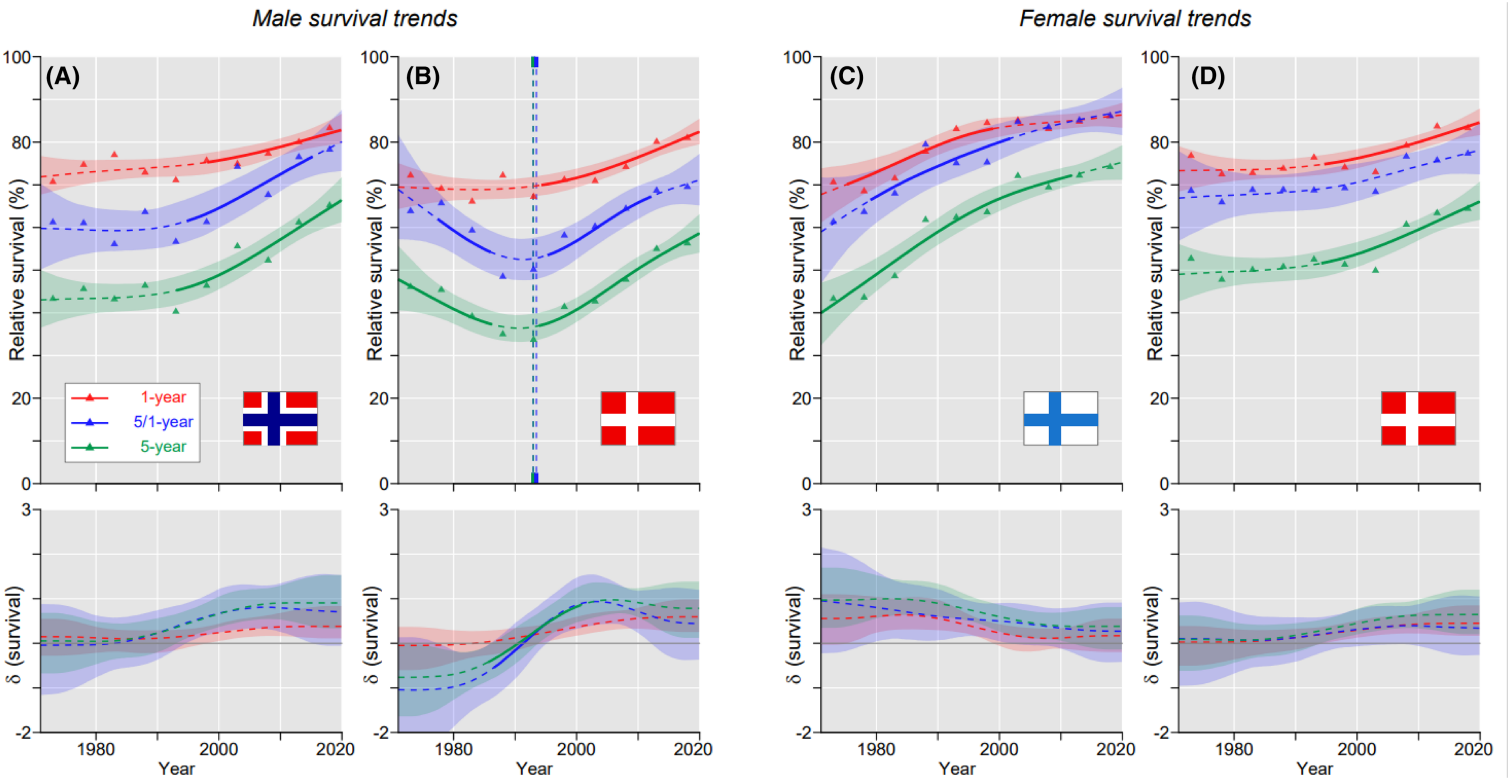
Oral cancer: relative 1‐, 5/1‐ and 5‐year survival in Norwegian (A) and Danish men (B), and in Finnish (C), and Danish women (D). The vertical lines mark a detectable change in the survival trends (“breakpoints”) and the bottom curves show estimated annual changes in survival. The curves are solid if there is >95% plausibility that the curve grows or declines. Shadow areas indicate 95% credible interval. All curves are color coded (see the insert).

The best 5‐year survival for male oral cancer was in NO (65.2%) and the worst country was DK (56.3%); for women the best country was FI (74.2%) and the worst was DK (64.4%, the difference to FI was significant). Data on these countries are presented in Figure 1 for trends of the three survival metrics through the 50‐year period. For NO men 1‐year survival increased linearly while 5/1‐ and 5‐year survival remained initially stable but shot up after 1990 (Figure 1A). For DK men in Figure 1B 5/1‐ and 5‐year survival curves described a V‐shaped paths, with initial decrease until 1995 and recovery thereafter. Survival curves for FI women increased steeply from the beginning on but the slopes started to decline at around 2000; 1‐ and 5/1‐ years curves merged towards the end (Figure 1C). The poor performance for DK as compared to FI women was explained by the slow initial increase in all survival curves (Figure 1D). Survival curves for the other countries are shown in Figure S1. All curves showed almost linear increases, except that for SE men initial 5/1‐ and 5‐years curves decreased.

**FIGURE 2:**
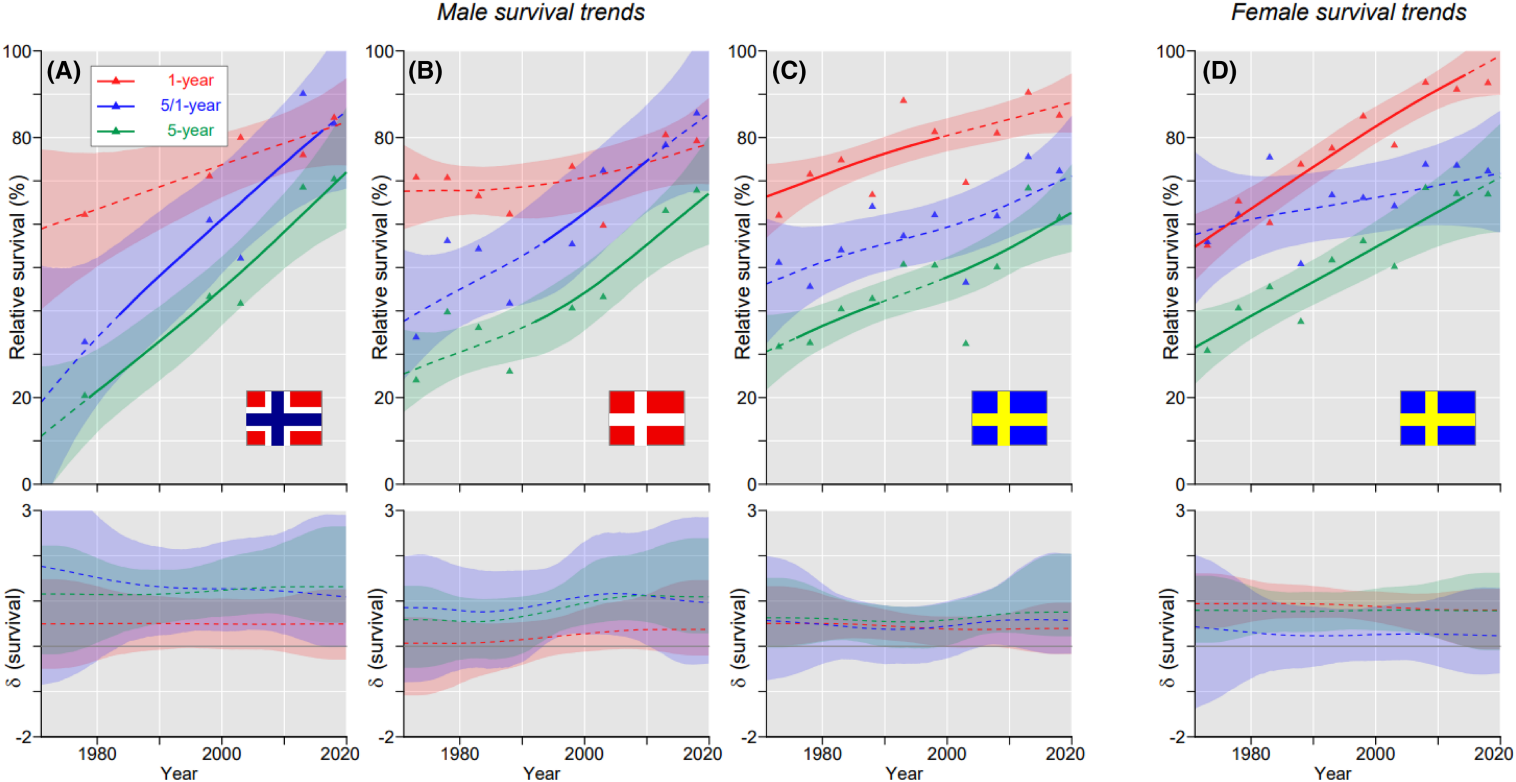
Nasopharyngeal cancer: relative 1‐, 5/1‐ and 5‐year survival in Norwegian (A), Danish (B), and Swedish men (C), and Swedish women (D). The bottom curves show estimated annual changes in survival. The curves are solid if there is >95% plausibility that the curve grows or declines. Shadow areas indicate 95% credible interval. All curves are color coded (see the insert).

For the rare nasopharyngeal cancer, complete survival data (missing datapoints) in 5‐year periods were only available for men in NO, with the best survival (70.4%), DK (67.8%) and SE (61.5%), and for women for SE only (66.9%) (Figure 2). For men the performance correlated with the steepness of the slopes in all the survival metrics, NO>DK > SE. For NO men, 1‐year and 5/1‐year survival curves met each other, whereas for SE women they diverged with time. In Table S1, 5‐year survival data for nasopharyngeal cancer are given in 10‐year intervals and NO survival was best for men (73.8%) and FI for women (77.4%).

**FIGURE 3:**
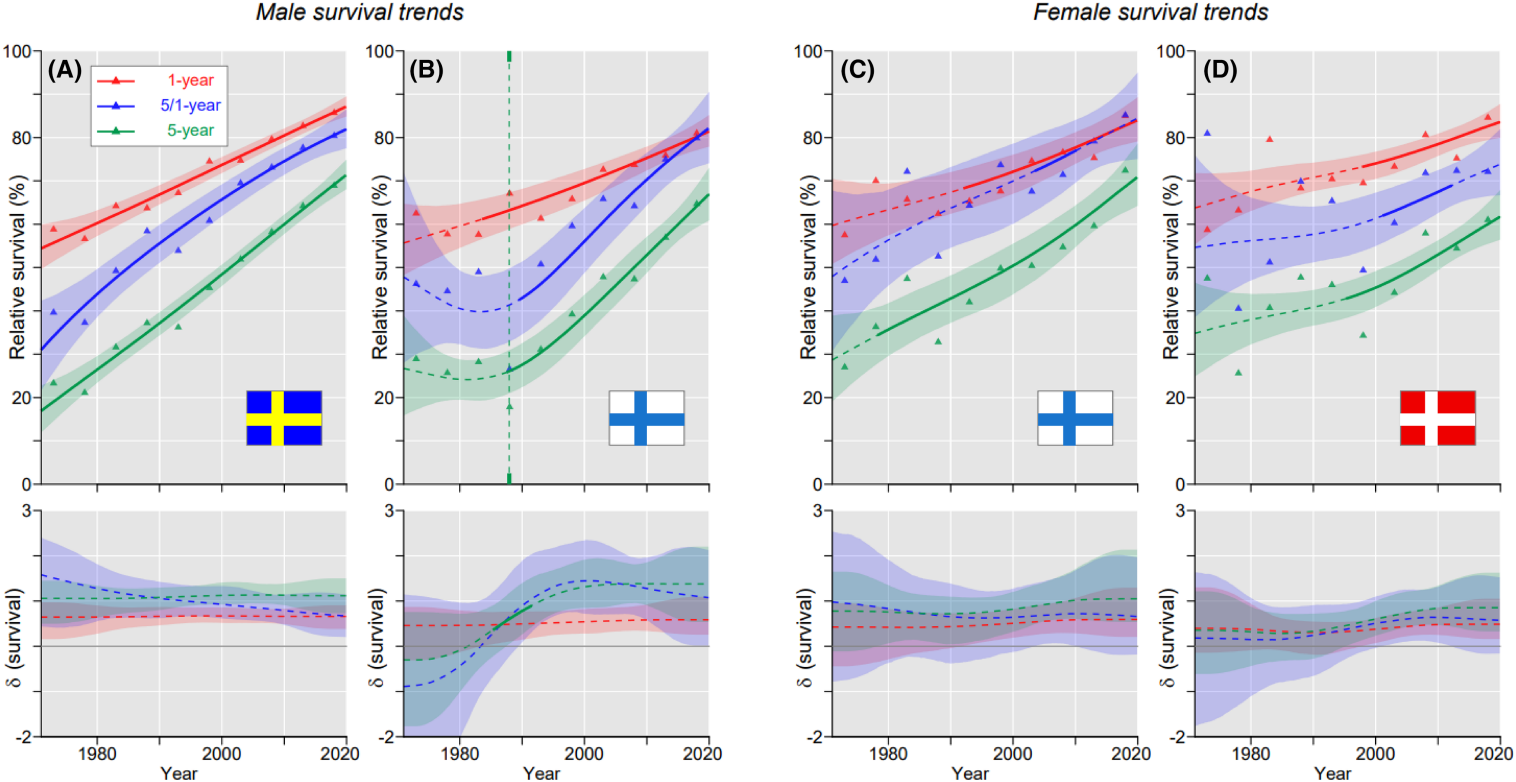
Oropharyngeal cancer: relative 1‐, 5/1‐ and 5‐year survival in Swedish (A) and Finnish men (B), and in Finnish (C) and Danish women (D). The vertical lines mark a detectable change in the survival trends (“breakpoints”) and the bottom curves show estimated annual changes in survival. The curves are solid if there is >95% plausibility that the curve grows or declines. Shadow areas indicate 95% credible interval. All curves are color coded (see the insert).

For male oropharyngeal cancer, SE had the best (68.9%) and FI males the worst (64.7%) 5‐year survival Figure 3A,B. All survival curves increased almost linearly for SE but for FI the linear increase started in 1990 and 5/1 year curve caught up with 1‐year curve at the end. For women, FI showed the best 5‐year survival (72.4%) and DK the worst (61.0%) Figure 3C,D. In FI the survival curves were close to linear while in DK there was a slow phase until year 2000. Among the other countries there were steady increases, except that for NO men and SE women initial 5/1‐ and 5‐years curves lagged behind (Figure S2).

**FIGURE 4:**
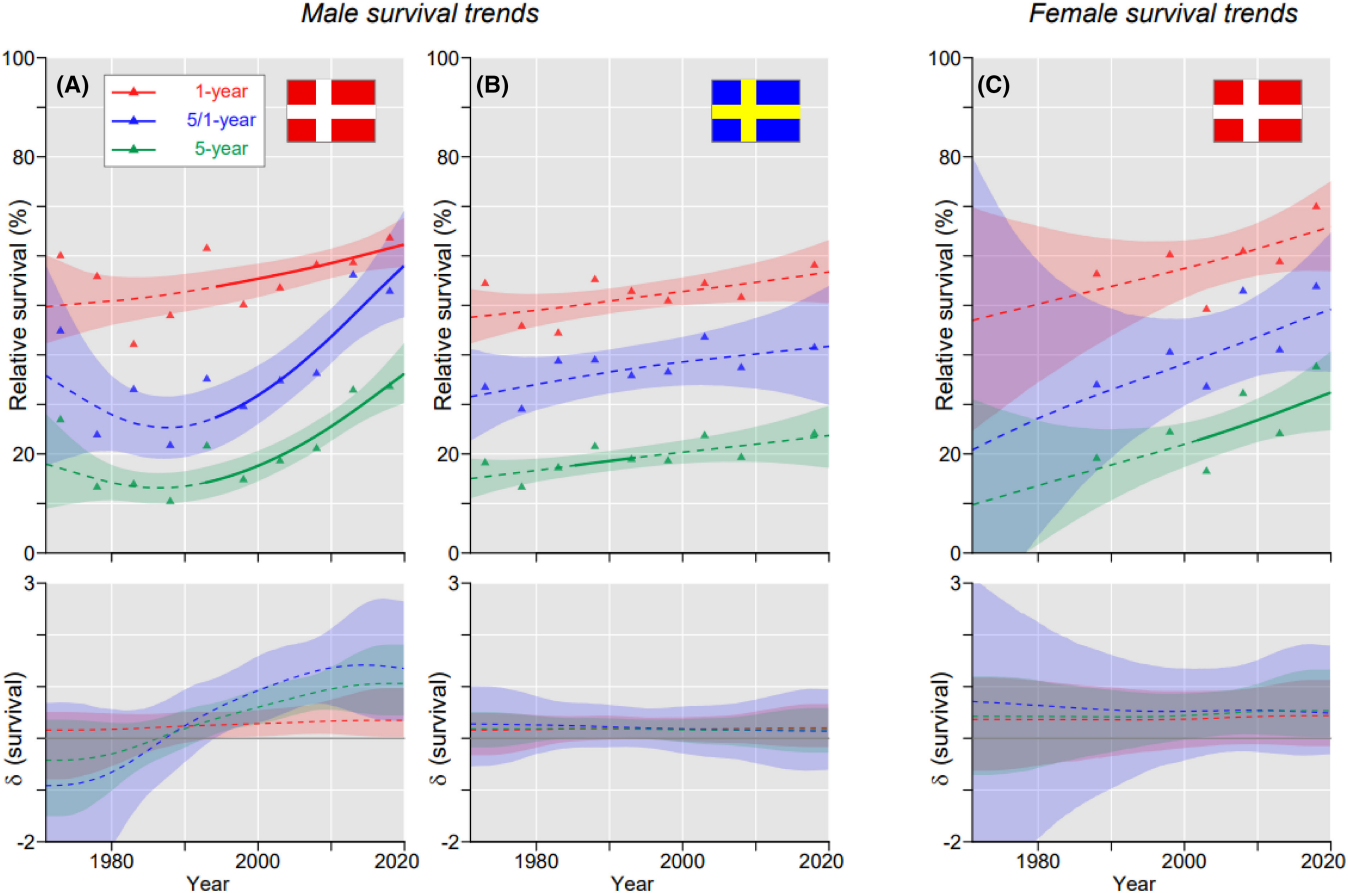
Hypopharyngeal cancer: relative 1‐, 5/1‐ and 5‐year survival in Norwegian (A), and Swedish men (B), and in Danish women (C). The bottom curves show estimated annual changes in survival. The curves are solid if there is >95% plausibility that the curve grows or declines. Shadow areas indicate 95% credible interval. All curves are color coded (see the insert).

Data on hypopharyngeal cancers were based on small numbers and were not complete for all countries. Thus in Figure 4 we present male data for DK (final 5‐ year survival 33.6%) and SE (24.1%), and female data for DK (37.6%). While the SE male and DK female data showed modestly increasing linear graphs, DK male 5/1‐ and 5‐year survival started to improve steeply around year 2000. Considering survival in 10‐periods (Table S1), 5‐year survival data for hypopharyngeal cancer was best for NO men (34.4%) and for FI women (35.7%).

**FIGURE 5:**
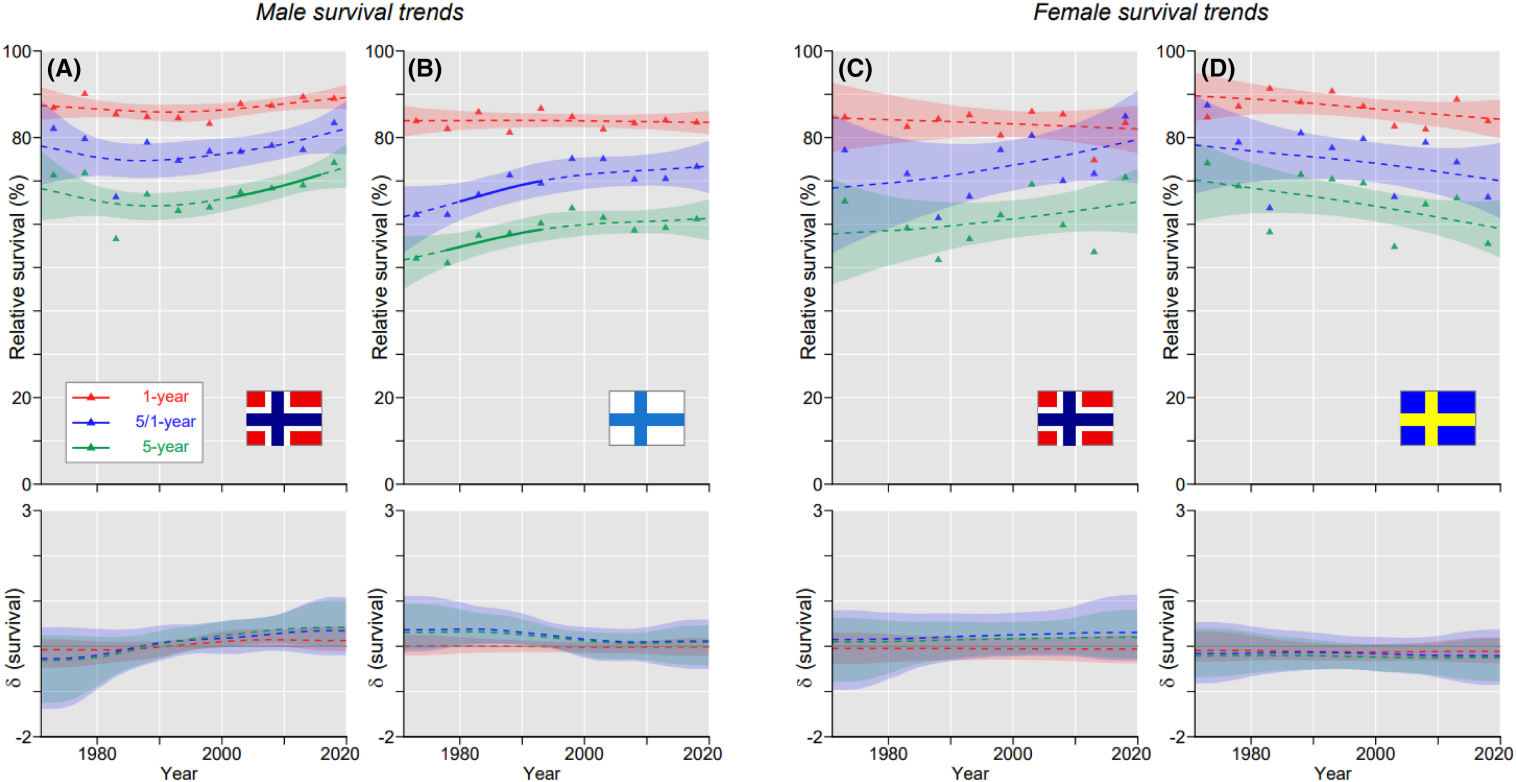
Laryngeal cancer: relative 1‐, 5/1‐ and 5‐year survival in Norwegian (A), and Finnish men (B), and in Norwegian (C), and Swedish women (D). The bottom curves show estimated annual changes in survival. The curves are solid if there is >95% plausibility that the curve grows or declines. Shadow areas indicate 95% credible interval. All curves are color coded (see the insert).

For laryngeal cancer the best and the worst male 5‐year survival was for NO (74.2%) and FI (61.2%, significantly below NO survival), and for women (FI data lacking) these were for NO (70.8%) and SE (55.5%) (Figure 5). Survival graphs were flat considering the wide credible intervals. They were also flat for the other countries (Figure S3).

In the US SEER database 5‐year survival for oropharyngeal and tonsil cancer in Non‐Hispanic White men was 76.2% and in women it was 69.1% in years 2012–18. None of the other cancers of the present interest were included.

Table S1 can be used to calculate the mean 5‐year survival in the last period, which was 61.6% for male and 68.6% for female oral cancer, 65.5% for male and 67.6% for female nasopharyngeal cancer (in 10‐year periods), 66.3% for male and 67.4% for female oropharyngeal cancer, 29.5% for male and 31.6% for female hypopharyngeal cancer (in 10‐year periods) and 68.9% for male and 65.2% female oral cancer (female data for FI missing).

The table enables also assessment of the improvement in 5‐year survival over the 50‐year period. For oral cancer by far the largest improvement took place in FI, 33.6% units for men and 30.9% units for women. Survival in FI men more than doubled in 50 years. For NO men survival in nasopharyngeal cancer more than doubled and their survival increased by 53.6% units (measured at 10‐year periods). For female survival FI was the top improver with 46.3% units. For male oropharyngeal cancer, NO and SE had a tie (45.2 and 45.1% units, respectively). On the female survival, FI was an overwhelming winner, 45.4% units, close to trebling of survival. For hypopharyngeal cancer survival, NO men achieved a 20.6% point increase compared to 24.8% points for FI women. For laryngeal cancer, survival for FI men increase by 9.1% points, and DK women also improved survival by 12.7% units while survival for SE women decreased by 18.6% units.

Table S1 invites also to the comparison of survival by sex. The only cancer for which the last 5‐year survival showed some sex preferences was oral cancer in which female survival was better than male survival in every country but the only significant difference was in FI.

In Table S2, 1‐ and 5/1‐ year survival is tabulated next to each other to enable comparison in survival between the first year and subsequent 4 years after diagnosis. For oral cancers all 1‐year survival rates exceeded those of 5/1‐year survival rates, except that in the last periods for FI women the latter were marginally better, implying that equally many patients died in the first year as in the subsequent 4 years. For nasopharyngeal cancer in FI and NO men and women and in DK women the last 5/1‐ years survival rates exceeded those for year 1. For oropharyngeal cancer this was also observed for FI and NO women and for laryngeal cancer for NO women. For nasopharyngeal and oropharyngeal cancer some penultimate periods also showed this change.

## 4 DISCUSSION

The aim of the study was to describe the past and the current survival experience in NH cancers in the four Nordic countries. This was accomplished thanks to the NORDCAN database maintained by the local cancer registries. As the first novel finding we could observe that 1‐ and 5‐year survival in the best countries (which varied by the type of cancer) improved steadily over the 50‐year period; the worst countries stumbled in the early part of 5‐year survival, referred to as “V‐shaped survival” (cf. male and female survival for oral and oropharyngeal cancers, Figure 1,, 3). Most importantly, 1‐year survival developed well for all cancers (but laryngeal cancer) and countries while 5‐year survival initially plunged in the countries of poor performance. This could suggest that the initial treatment was successful but it had untoward late sequelae which were overlooked or intractable. Alternatively, there might have been some fundamental changes in oncological practice that turned out to be unsuccessful. There was a curious contrast for oropharyngeal cancer in FI (Figure 3) as the initial 5‐year survival was suppressed only in men. The other novel observation revealed excellent final conditional survival between years 2 and 5; during these 4 years the number of deaths was lower than in year 1. This was observed in nasopharyngeal cancer in FI and NO patients and in DK women, and also in FI and/or NO women in oral, oropharyngeal and laryngeal cancers. These changes were found towards the end of the follow up with a statistical implication that for these cancers 5‐year survival was increasingly driven by 1‐year survival (more deaths in year 1 than in years 2–5). The clinical implication is that diagnostics and treatment were able to better prevent cancer deaths in interval 2–5 years after diagnosis (probably early diagnosis enabled curative treatment) than in the first year (aggressive early metastases).

The up‐to‐data 5‐year survival data for the Nordic countries showed favorable development for all cancers but laryngeal cancer for which poor survival development has been noted before among all solid cancers.^30^ However as laryngeal cancer showed the highest survival in 1971–74, its 5‐year survival was at the 65% mark in 2016–20 together with oral, nasopharyngeal and oropharyngeal cancers; survival was lower, 30%, only for hypopharyngeal cancer. The reason for the deviant behavior of laryngeal cancer to the other HN cancers is not clear because the factors influencing survival in HN cancer appear to be largely shared.^31^ However it is also shared in US where 5‐year survival in laryngeal cancer has been stagnant since the mid‐1970s (see: https://seer.cancer.gov/statfacts/html/laryn.html). One possibility may be that early diagnosis is laryngeal cancer has not been as successful as in the other HN cancers.^32^ A 50‐year increase was about 30% units for nasopharyngeal and oropharyngeal cancers, 20% units for oral cancer and somewhat less for hypopharyngeal cancer, all of which are comparable with all solid cancers in these countries.^30^ In all HN cancers, the best 5‐year survival was over 70% with the exception of hypopharyngeal cancer for which best survival reached 35%. Country‐specific 5‐year survival improved often most for the countries starting at the low level, and at the end few significant differences between the countries were noted (female oral cancer, FI > DK and male laryngeal cancer NO>FI). Reasons for stagnant survival in laryngeal cancer are not well understood. During the last decades, there has been essential changes in the treatment strategies of patients with advanced laryngeal cancer: shift towards organ preservation avoiding radical surgery and substitution of induction chemotherapy for chemoradiotherapy.^33^ These changes might have depressed survival in patients with locally advanced cancer.

The only cancer with indication of sex‐preference was oral cancer for which all female 5‐year survival rates were nominally higher than the male ones, but only for FI the difference was significant. Dentists play a significant role in referring patients to tertiary clinics due to suspicious/malignant mucosal changes of the oral cavity. It can be hypothesized that since women take better care of their oral health with regular dental visits, their oral cancer might be diagnosed at earlier stage with better prognosis.^34^, ^35^ Lacking of sex‐preference is as such worth of consideration because sex‐specific incidence rates (and thus risk factors) were much higher for men in these cancers (Table 1). In survival there was a curious country‐specific sex‐bias. NO showed the best 5‐years survival in 4/5 male cancers but only in 1/5 female cancers (i.e., laryngeal cancer for which FI data were lacking). FI dominated female cancers, showing the highest 5‐year survival in all four cancers for which data were available, and FI male survival was worst for 3/5 cancers.

Whether the known risk factors for these cancers played a role cannot be answered based on the ecological data available. SE men with historically low smoking levels showed the best survival for oropharyngeal cancer but SE male patients are often HPV‐positive which would improve survival.^13^, ^36^, ^37^ DK men and women have shown historically high smoking and alcohol consumption levels and their survival rates were rather weak with no best positions.^23^

To put the Nordic survival figures in perspective, we searched data in the US SEER database, however only data for combined oropharyngeal and tonsil cancer were available; in years 2012–18 for Non‐Hispanic White men 5‐year survival was 76.2%, better than in the present Nordic data, and for women it was 69.1%, at the level of the present results. Recent European survival studies on HN cancers are not many, excluding a series of papers from DK covering a period between 1980 and 2014.^9^, ^38^, ^39^, ^40^, ^41^ EUROCARE‐5 published a comprehensive survival study on all HN cancers up to year 2008 from 86 cancer registries.^14^ For all Europe, 5‐ year survival in oropharyngeal cancer was 38.7%, in nasopharyngeal cancer it was 49.0% and in hypopharyngeal cancer it was 24.6%. In that study, the Nordic cancer registries showed the best survival in oropharyngeal cancer.^14^ Dutch 5‐year survival in 2007–2011 in oropharyngeal cancer was 48% and in hypopharyngeal cancer it was 33%.^42^ These data in Ireland in year 2010 were 56.5% and 36.7%, and additionally nasopharyngeal data was provided, 56.5%.^43^ A study from Estonia on 5‐year survival in 2010–2016 reported 44% and 63% survival for cancers of the oral cavity and larynx, and 24% and 17% for cancers of the oropharynx and hypopharynx.^44^

In HN cancers, the current treatment approach is designed for every individual patient by a multidisciplinary team considering anatomical subsite, stage, patient characteristics, comorbidities, functional considerations, local expertize and patient wishes.^45^, ^46^ HPV status is considered for oropharyngeal cancer but it has no main effect on the treatment plan.^45^ Treatment modalities include surgery, radiation or chemotherapy or their combinations of which surgery was the main method historically.^4^ For localized cancer (stage I and II), surgery alone or radiotherapy alone may be curative; laser microsurgery for oropharyngeal and hypopharyngeal cancer preserve function and intensity modulated and image‐guided radiotherapy may increase survival.^45^ For localized nasopharyngeal cancer intensity modulated radiotherapy is the treatment of choice which is supplemented with chemotherapy in high‐risk patients.^47^ Most patients have locally advanced or metastatic cancer for which cisplatin‐based chemotherapy is applied in combination with intensity modulated radiotherapy, or chemotherapy may be applied in adjuvant setting or as an induction chemotherapy.^47^ For nodal metastases selected targeted radiation saves adjacent structures.^48^ The rapidly increasing oropharyngeal cancer is becoming a major clinical challenge and the predicted relief by HPV vaccination will be gradual with major impact several decades into the future.^49^, ^50^, ^51^, ^52^ In the meanwhile the focus should be in avoidance of infections and in development of effective screening programs for detection of precursor lesions for oropharyngeal cancer.^52^

The inherent limitation of a database that spans 50‐years of data is the lack of many details, such as clinical characteristics of the patients, their risk factors (no HPV data are available) and the applied treatment. If we attempted to consider these, we could use known ecological data. The strength of NORDCAN is to offer high‐level diagnostic data for the complete population of four countries, thus providing a unique “real world” view of cancer experience from the past to the present. In the case of HN cancer, the prevalence of many risk factors is known and the contrasts in exposures between the Nordic countries are probably wider than one could expected in close neighbors.

In conclusion, we showed overall a positive development in 1‐ and 5‐year survival for all HN cancers excluding laryngeal cancer. Survival in laryngeal cancer was however best among HN cancers 50 years ago and even though hardly any improvement has taken place since then survival was currently at the level of the best HN cancers; laryngeal cancer has been an underperformer compared to most other solid cancer in the Nordic countries. Analysis of conditional survival showed that for nasopharyngeal cancer and for some other cancers in FI and NO women 5/1‐year survival matched 1‐year survival in the last period, implying that no more patients died in 4 years (years 2–5) than in the first year after diagnosis. Treatment modalities of surgery, radiotherapy and chemotherapy have improved and an increasing consideration has been given to preservation of organ function.

## AUTHOR CONTRIBUTIONS

**Frantisek Zitricky:** Formal analysis (lead); investigation (lead); writing – original draft (supporting); writing – review and editing (equal). **Anni I. Koskinen:** Investigation (supporting); writing – review and editing (equal). **Otto Hemminki:** Investigation (supporting); writing – review and editing (equal). **Asta Försti:** Investigation (supporting); writing – review and editing (equal). **Akseli Hemminki:** Investigation (supporting); writing – review and editing (equal). **Kari Hemminki:** Conceptualization (lead); formal analysis (lead); investigation (lead); supervision (lead); writing – original draft (lead).

## FUNDING INFORMATION

Supported by the European Union's Horizon 2020 research and innovation programme, grant No 856620, Jane and Aatos Erkko Foundation, Sigrid Juselius Foundation, Finnish Cancer Organizations, University of Helsinki, Helsinki University Central Hospital, Novo Nordisk Foundation, Päivikki and Sakari Sohlberg Foundation, Finnish Red Cross Blood Service, the Cooperatio Program, research area SURG and National Institute for Cancer—NICR (Programme EXCELES, ID Project No. LX22NPO5102), funded by the European Union—Next Generation EU.

## CONFLICT OF INTEREST STATEMENT

A.H. is shareholder in Targovax ASA. A.H. is employee and shareholder in TILT Biotherapeutics Ltd. Other authors declared no conflict of interest.

## ETHICS STATEMENT

Aggregated data from a publically accessible database were used posing no ethical issues.

## CONSENT

Not applicable.

## Supporting information


Data S1.

## Data Availability

Publically available NORDCAN data can be accessed at (https://NORDCAN.iarc.fr/en/database#bloc2).
