# Supplementary material for: Survival in oral and pharyngeal cancers is catching up with laryngeal cancer in the NORDIC countries through a half century
Source: Cancer Med. 2024 Jan 2;13(1):e6867. doi: 10.1002/cam4.6867 (PMC10807619; doi:10.1002/cam4.6867)
Supplement: Supplementary file 1 — Data S1. [file CAM4-13-e6867-s001.docx]

**SUPPLEMENTARY MATERIAL**

| Male cancers | | | | | Female cancers |  |  |  |
| --- | --- | --- | --- | --- | --- | --- | --- | --- |
| Oral cavity | Denmark | Finland | Norway | Sweden | Denmark | Finland | Norway | Sweden |
| 1971-1975 | 46.1[38.8-54.9] | 28.6[19.0-43.1] | 43.3[36.4-51.4] | 47.4[42.0-53.4] | 52.7[44.9-61.9] | 43.3[34.9-53.7] | 52.4[43.6-63.1] | 56.6[51.0-62.9] |
| 1976-1980 | 45.4[38.7-53.2] | 39.9[30.6-52.0] | 45.6[38.7-53.6] | 45.3[40.0-51.4] | 47.8[41.5-55.1] | 43.6[36.5-52.0] | 56.8[49.0-65.9] | 55.6[50.1-61.6] |
| 1981-1985 | 39.2[33.3-46.2] | 43.3[34.9-53.7] | 43.2[36.9-50.6] | 38.1[33.7-43.0] | 50.1[44.3-56.7] | 48.6[41.3-57.1] | 52.3[45.3-60.4] | 54.5[49.4-60.3] |
| 1986-1990 | 35.0[29.7-41.1] | 41.3[32.0-53.3] | 46.4[40.1-53.7] | 42.4[37.5-48.0] | 50.8[45.2-57.2] | 61.8[54.9-69.7] | 57.4[50.8-64.8] | 52.8[48.1-58.1] |
| 1991-1995 | 33.7[28.4-40.1] | 49.0[41.4-58.0] | 40.3[34.6-46.8] | 48.3[44.0-53.0] | 52.5[46.9-58.7] | 62.4[56.4-68.9] | 62.7[56.0-70.3] | 58.8[54.1-63.9] |
| 1996-2000 | 41.4[36.4-47.2] | 48.5[40.6-57.9] | 46.4[40.6-52.9] | 49.8[45.2-54.8] | 51.3[46.0-57.2] | 63.6[57.5-70.2] | 62.4[56.0-69.5] | 59.9[55.3-64.9] |
| 2001-2005 | 42.7[37.4-48.6] | 47.1[40.7-54.6]* | 55.6[49.0-63.0] | 50.4[45.9-55.2] | 49.9[44.7-55.7]* | 72.1[67.3-77.2] | 63.8[57.6-70.6] | 61.5[57.2-66.1] |
| 2006-2010 | 47.8[43.2-52.9] | 60.3[54.9-66.2] | 52.3[46.5-58.9] | 54.7[50.7-59.1] | 60.7[55.9-65.8] | 69.4[64.9-74.1] | 65.0[59.4-71.2] | 64.3[60.4-68.5] |
| 2011-2015 | 55.0[50.7-59.6] | 61.8[57.1-66.9] | 61.2[56.0-67.0] | 62.8[59.1-66.8] | 63.4[58.8-68.4] | 72.2[68.2-76.5] | 70.3[64.9-76.1] | 68.6[65.0-72.4] |
| 2016-2020 | 56.3[52.3-60.6] | 62.2[57.8-67.0] | 65.2[60.2-70.6] | 62.7[59.1-66.4] | 64.4[60.2-68.9] | 74.2[70.3-78.4] | 66.6[61.4-72.1] | 69.0[65.6-72.6] |
| Nasopharynx | | | | | | | | |
| 1971-1980 | 33.0[24.7-44.0] | 33.7[22.7-50.1] | 20.2[12.8-31.9] | 33.3[26.7-41.4] | 42.8[30.2-60.7] | 31.1[21.0-46.0] | 34.4[22.5-52.7] | 35.6[28.2-44.9] |
| 1981-1990 | 32.4[24.3-43.3] | 40.1[27.6-58.1] | 25.7[17.6-37.5] | 41.5[34.7-49.7] | 19.3[10.0-37.1] | 36.2[24.7-53.3] | 45.8[30.5-68.9] | 44.1[35.7-54.4] |
| 1991-2000 | 29.5[19.6-44.2] | 39.8[27.6-57.2] | 41.0[28.6-58.6] | 50.6[41.7-61.5] | 40.9[28.6-58.3] | 53.5[39.7-72.3] |  | 54.6[43.7-68.2] |
| 2001-2010 | 46.1[35.3-60.4] | 50.0[38.2-65.5] | 46.8[34.5-63.4] | 43.6[34.4-55.1] | 42.5[28.1-64.3] | 42.0[26.0-68.0] | 44.2[27.3-71.7] | 53.9[41.5-70.0] |
| 2011-2020 | 64.8[52.6-79.7] | 58.9[45.6-76.1] | 73.8[59.8-91.0] | 64.6[54.9-76.1] | 47.6[33.9-66.8] | 77.4[62.3-96.0] | 73.7[54.7-99.4] | 71.7[57.6-89.3] |
| Oropharynx | | | | | | | | |
| 1971-1975 | 26.1[17.3-39.3] | 28.9[18.1-46.3] | 20.9[13.0-33.7] | 23.3[15.4-35.1] | 47.5[33.2-67.9] | 27.0[13.9-52.5] |  | 48.5[36.4-64.6] |
| 1976-1980 | 23.8[17.5-32.3] | 25.7[17.6-37.8] | 19.1[10.5-34.8] | 21.1[15.5-28.6] | 25.6[14.1-46.1] | 36.3[24.7-53.4] | 35.6[22.3-56.9] | 45.5[36.1-57.2] |
| 1981-1985 | 27.2[19.1-38.9] | 28.2[18.7-42.6] | 21.4[13.4-34.3] | 31.6[25.4-39.4] | 40.7[30.8-53.8] | 47.4[35.8-62.7] | 39.6[27.2-57.5] | 30.9[22.5-42.4] |
| 1986-1990 | 34.5[27.8-42.9] | 17.8[10.4-30.3] | 23.7[13.7-41.3] | 37.2[31.1-44.5] | 47.7[38.3-59.4] | 32.8[18.2-59.2] | 39.7[27.5-57.5] | 46.1[37.3-57.1] |
| 1991-1995 | 29.3[23.9-36.0] | 31.1[21.0-45.9] | 19.3[13.6-27.4]* | 36.2[29.7-44.1] | 46.0[37.5-56.4] | 42.0[29.0-60.9] | 38.7[26.2-57.2] | 50.2[42.2-59.7] |
| 1996-2000 | 35.8[29.9-42.8] | 39.2[27.9-55.1] | 43.0[33.8-54.6] | 45.3[39.4-52.2] | 34.3[27.0-43.6] | 49.8[36.3-68.2] | 39.1[29.7-51.4] | 52.3[44.7-61.3] |
| 2001-2005 | 39.7[33.9-46.5] | 47.8[39.1-58.5] | 38.2[31.4-46.5] | 51.9[46.3-58.2] | 44.2[36.5-53.4] | 50.4[36.8-68.9] | 55.0[43.9-68.9] | 51.2[43.9-59.8] |
| 2006-2010 | 51.1[45.7-57.2] | 47.3[39.2-56.9] | 49.8[42.8-58.0] | 58.2[53.3-63.5] | 57.9[50.1-67.0] | 54.7[43.5-68.7] | 52.9[43.4-64.6] | 60.6[53.7-68.3] |
| 2011-2015 | 58.1[53.5-63.2] | 56.9[50.2-64.5] | 60.7[53.9-68.4] | 64.2[60.0-68.6] | 54.4[48.3-61.3] | 59.6[51.2-69.4] | 57.6[48.5-68.5] | 72.0[66.4-78.1] |
| 2016-2020 | 65.6[61.6-69.9] | 64.7[59.2-70.7] | 66.1[60.0-72.8] | 68.9[65.2-72.8] | 61.0[55.6-66.9] | 72.4[65.2-80.3] | 65.1[56.8-74.6] | 71.1[65.8-76.7] |
| Hypopharynx | | | | | | | | |
| 1971-1980 | 19.6[13.0-29.5] | 11.0[5.8-20.6] | 13.8[9.2-20.7] | 14.1[10.8-18.5] | 19.5[10.3-37.1] | 10.9[5.9-20.0] | 25.3[15.8-40.5] | 17.8[12.5-25.4] |
| 1981-1990 | 11.9[7.9-18.0] | 11.0[6.1-19.8] | 17.2[12.3-24.2] | 17.7[14.4-21.8] | 16.2[8.7-30.4] | 20.5[12.3-34.2] | 33.9[22.4-51.4] | 13.3[8.3-21.3] |
| 1991-2000 | 17.2[12.4-23.8] | 17.4[11.9-25.4] | 22.5[17.0-29.7] | 18.9[15.4-23.1] | 22.0[14.7-33.0] | 18.9[10.4-34.5] | 7.0[2.3-20.9] | 27.7[20.1-38.2] |
| 2001-2010 | 19.9[16.2-24.4]* | 27.9[21.7-36.0] | 20.2[14.9-27.2] | 20.1[16.3-24.7] | 26.0[19.1-35.5] | 21.9[11.7-41.0] | 27.0[17.1-42.7] | 30.1[22.8-39.6] |
| 2011-2020 | 32.8[28.8-37.4] | 27.5[21.5-35.0] | 34.4[26.6-44.5] | 23.3[18.5-29.3] | 32.3[24.7-42.2] | 35.7[21.2-60.0] | 24.6[13.3-45.8] | 33.6[24.7-45.7] |
| Larynx | | | | | | | | |
| 1971-1975 | 72.0[65.8-78.7] | 52.1[44.4-61.2] | 71.3[63.0-80.8] | 67.8[62.2-73.9] | 56.7[45.6-70.4] | 52.5[34.5-79.8] | 65.3[47.0-90.8] | 74.1[54.9-100.0] |
| 1976-1980 | 55.7[50.7-61.3] | 51.0[45.1-57.7] | 71.8[64.4-80.1] | 63.7[58.7-69.2] | 63.2[51.5-77.6] |  |  | 68.8[56.0-84.5] |
| 1981-1985 | 59.0[54.1-64.2] | 57.4[49.8-66.1] | 56.6[50.4-63.6] | 66.8[62.4-71.5] | 62.7[53.2-73.9] | 61.1[47.2-79.0] | 59.1[45.8-76.4] | 58.2[47.6-71.2] |
| 1986-1990 | 57.5[52.7-62.9] | 57.9[51.9-64.5] | 66.9[61.0-73.3] | 68.4[64.0-73.1] | 51.9[44.5-60.6] | 44.5[32.2-61.5] | 51.8[35.6-75.4] | 71.5[60.7-84.1] |
| 1991-1995 | 56.6[52.0-61.6] | 60.2[54.0-67.1] | 63.1[56.8-70.0] | 68.1[63.6-73.0] | 54.5[46.8-63.4] |  | 56.6[45.0-71.2] | 70.4[61.1-81.1] |
| 1996-2000 | 61.8[57.3-66.6] | 63.7[57.3-70.8] | 63.9[58.3-70.0] | 68.3[63.6-73.5] | 50.0[42.1-59.5] |  | 62.1[51.9-74.2] | 69.5[60.0-80.4] |
| 2001-2005 | 57.4[52.7-62.4] | 61.5[55.7-68.0] | 67.4[62.1-73.1] | 66.0[61.6-70.6] | 60.8[52.5-70.5] |  | 69.2[58.1-82.5] | 54.8[46.3-64.7] |
| 2006-2010 | 64.5[60.4-69.0] | 58.6[53.1-64.6] | 68.3[62.9-74.0] | 69.7[65.2-74.4] | 58.4[50.8-67.0] | 67.8[55.4-83.0] | 59.8[48.5-73.8] | 64.6[56.3-74.2] |
| 2011-2015 | 65.8[61.5-70.4] | 59.2[53.6-65.5] | 69.0[63.9-74.5] | 70.4[65.8-75.4] | 59.1[51.4-67.9] |  | 53.6[42.8-67.2] | 66.0[57.1-76.3] |
| 2016-2020 | 69.4[65.3-73.7] | 61.2[56.0-66.9] | 74.2[69.2-79.6] | 70.8[66.6-75.2] | 69.4[61.4-78.4] |  | 70.8[61.4-81.7] | 55.5[46.9-65.7] |

**Supplementary Table 1**. 5-year relative survival [95% confidence interval] for specific cancer sites from 1971 to 2020. *Significant increase between marked and the next period.

**Supplementary Table 2**. 1-year relative survival [95% confidence interval] and 5/1-year conditional survival for specific cancer sites from 1971 to 2020. *Significant increase in 1-year survival between the marked and the next period.

| Male cancers | | | | | Female cancers |  |  |  |
| --- | --- | --- | --- | --- | --- | --- | --- | --- |
| Oral cavity 1-year | Denmark | Finland | Norway | Sweden | Denmark | Finland | Norway | Sweden |
| 1971-1975 | 72.2[66.1-78.8] | 62.8[51.9-75.9] | 70.7[65.1-76.8] | 69.9[65.8-74.3] | 76.8[70.7-83.3] | 70.6[63.1-78.9] | 76.8[70.4-83.8] | 75.5[71.2-80.0] |
| 1976-1980 | 69.1[63.9-74.9] | 71.5[63.8-80.2] | 74.7[69.3-80.6] | 68.5[64.4-72.9] | 72.5[67.4-78.1] | 68.5[62.0-75.7] | 81.2[75.7-87.1] | 77.2[73.1-81.6] |
| 1981-1985 | 66.1[61.2-71.5] | 68.2[61.0-76.3] | 77.0[72.4-81.8] | 68.9[65.2-72.8] | 72.8[68.0-77.8] | 71.5[65.4-78.3] | 75.3[69.6-81.4] | 77.4[73.4-81.6] |
| 1986-1990 | 72.2[67.8-77.0] | 79.9[73.4-86.9] | 72.9[68.1-78.1] | 73.7[70.0-77.5] | 73.8[69.3-78.6] | 77.8[72.5-83.6] | 76.5[71.3-82.0] | 80.9[77.5-84.5] |
| 1991-1995 | 67.2[62.7-71.9] | 75.5[69.7-81.9] | 71.1[66.5-76.1] | 74.2[70.9-77.7] | 76.4[72.0-81.0] | 83.1[79.1-87.3] | 80.2[75.3-85.4] | 80.9[77.5-84.4] |
| 1996-2000 | 71.2[66.9-75.7] | 79.2[74.2-84.7] | 75.7[71.2-80.6] | 73.4[69.8-77.2] | 74.1[70.0-78.6] | 84.5[80.3-89.0] | 81.4[76.7-86.2] | 80.1[76.7-83.7] |
| 2001-2005 | 70.9[66.8-75.2] | 75.8[70.6-81.2] | 74.9[70.1-80.1] | 72.5[68.9-76.2] | 73.0[68.8-77.5] | 85.1[81.8-88.6] | 83.2[78.9-87.8] | 82.9[79.9-86.0] |
| 2006-2010 | 74.2[70.6-78.1] | 79.4[75.6-83.4] | 77.3[72.9-81.9] | 77.8[74.8-80.9] | 79.2[75.6-82.9] | 83.1[79.9-86.4] | 83.4[79.4-87.7] | 82.0[79.2-84.9] |
| 2011-2015 | 80.1[77.1-83.3] | 79.0[75.7-82.4] | 80.0[76.2-84.0] | 81.4[78.8-84.1] | 83.7[80.6-87.0] | 84.8[81.9-87.8] | 90.5[87.2-93.8] | 86.8[84.4-89.2] |
| 2016-2020 | 81.0[78.2-83.9] | 80.2[77.3-83.3] | 83.3[80.0-86.8] | 81.0[78.5-83.6] | 83.3[80.3-86.4] | 86.1[83.3-88.9] | 88.5[85.4-91.7] | 87.4[85.2-89.7] |
| 5/1-year | | | | | | | | |
| 1971-1975 | 63.9 | 45.5 | 61.2 | 67.8 | 68.6 | 61.3 | 68.2 | 75.0 |
| 1976-1980 | 65.7 | 55.8 | 61.0 | 66.1 | 65.9 | 63.6 | 70.0 | 72.0 |
| 1981-1985 | 59.3 | 63.5 | 56.1 | 55.3 | 68.8 | 68.0 | 69.5 | 70.4 |
| 1986-1990 | 48.5 | 51.7 | 63.6 | 57.5 | 68.8 | 79.4 | 75.0 | 65.3 |
| 1991-1995 | 50.1 | 64.9 | 56.7 | 65.1 | 68.7 | 75.1 | 78.2 | 72.7 |
| 1996-2000 | 58.1 | 61.2 | 61.3 | 67.8 | 69.2 | 75.3 | 76.7 | 74.8 |
| 2001-2005 | 60.2 | 62.1 | 74.2 | 69.5 | 68.4 | 84.7 | 76.7 | 74.2 |
| 2006-2010 | 64.4 | 75.9 | 67.7 | 70.3 | 76.6 | 83.5 | 77.9 | 78.4 |
| 2011-2015 | 68.7 | 78.2 | 76.5 | 77.1 | 75.7 | 85.1 | 77.7 | 79.0 |
| 2016-2020 | 69.5 | 77.6 | 78.3 | 77.4 | 77.3 | 86.2 | 75.3 | 78.9 |
| Nasopharynx 1-year | | | | | | | | |
| 1971-1980 | 70.4[62.1-79.9] | 65.7[53.2-81.0] | 56.7[45.4-70.9] | 67.6[61.3-74.7] | 70.8[58.1-86.2] | 52.6[41.2-67.3] | 62.2[49.3-78.7] | 59.2[52.1-67.2] |
| 1981-1990 | 66.6[58.8-75.5] | 70.7[58.9-84.9] | 67.2[57.1-79.1] | 71.5[65.6-78.0] | 67.5[54.1-84.1] | 64.0[51.8-79.2] | 70.0[55.5-88.2] | 67.9[60.2-76.6] |
| 1991-2000 | 67.1[56.2-80.1] | 69.8[59.0-82.7] | 68.2[57.0-81.6] | 83.9[77.7-90.5] | 75.8[64.7-88.8] | 71.6[59.3-86.4] |  | 80.5[71.7-90.4] |
| 2001-2010 | 69.6[59.9-81.0] | 72.1[61.7-84.2] | 82.9[72.5-94.8] | 76.3[68.5-85.0] | 75.6[62.2-91.9] | 77.7[62.4-96.7] | 62.2[44.8-86.3] | 81.6[72.8-91.5] |
| 2011-2020 | 81.6[73.4-90.8] | 73.4[62.0-86.8] | 83.7[74.9-93.6] | 88.0[82.1-94.3] | 67.0[53.0-84.7] | 85.7[73.2-100.3] | 85.1[72.0-100.6] | 91.7[83.8-100.3] |
| 5/1-year | | | | | | | | |
| 1971-1975 | 46.9 | 51.3 | 35.6 | 49.3 | 60.5 | 59.1 | 55.3 | 60.1 |
| 1976-1980 | 48.6 | 56.7 | 38.2 | 58.0 | 28.6 | 56.6 | 65.4 | 64.9 |
| 1981-1985 | 44.0 | 57.0 | 60.1 | 60.3 | 54.0 | 74.7 |  | 67.8 |
| 1986-1990 | 66.2 | 69.3 | 56.5 | 57.1 | 56.2 | 54.1 | 71.1 | 66.1 |
| 1991-1995 | 79.4 | 80.2 | 88.2 | 73.4 | 71.0 | 90.3 | 86.6 | 78.2 |
| Oropharynx 1-year | | | | | | | | |
| 1971-1975 | 55.5[45.5-67.7] | 62.5[50.4-77.6] | 55.6[46.6-66.3] | 58.8[50.6-68.4] | 58.7[45.7-75.4] | 57.5[41.8-79.2] |  | 70.1[60.4-81.5] |
| 1976-1980 | 60.6[53.3-69.0] | 57.7[47.2-70.5] | 54.2[43.0-68.4] | 56.6[50.1-64.0] | 63.2[53.3-75.1] | 70.0[57.9-84.6] | 67.0[54.3-82.6] | 67.3[58.9-76.9] |
| 1981-1985 | 63.7[56.8-71.3] | 57.6[47.4-70.1] | 61.7[52.9-72.0] | 64.2[58.1-71.0] | 79.5[72.0-87.8] | 65.7[54.8-78.8] | 77.9[66.5-91.3] | 70.0[61.6-79.5] |
| 1986-1990 | 59.8[53.8-66.4] | 67.1[56.8-79.2] | 57.2[47.3-69.2] | 63.7[58.4-69.4] | 68.3[60.0-77.9] | 62.4[48.9-79.7] | 74.6[63.3-88.1] | 74.1[66.1-83.0] |
| 1991-1995 | 55.2[49.5-61.7] | 61.3[51.1-73.5] | 54.2[46.6-63.0] | 67.2[61.6-73.3] | 70.4[63.4-78.3] | 65.3[52.7-80.8] | 76.0[66.0-87.5] | 72.0[65.3-79.4] |
| 1996-2000 | 66.5[61.3-72.2] | 65.8[54.5-79.6] | 69.2[61.7-77.5] | 74.5[69.7-79.6] | 69.5[62.6-77.3] | 67.6[54.5-84.0] | 66.0[56.9-76.6]* | 76.7[70.3-83.7] |
| 2001-2005 | 66.7[61.8-72.0] | 72.6[64.0-82.3] | 65.5[58.4-73.5] | 74.7[70.4-79.4] | 73.3[66.7-80.5] | 74.6[63.9-87.1] | 85.4[77.9-93.5] | 74.2[68.0-80.9] |
| 2006-2010 | 72.4[67.7-77.5] | 73.7[66.0-82.4] | 75.1[69.3-81.4] | 79.6[75.7-83.6] | 80.6[75.3-86.2] | 76.6[67.2-87.3] | 75.0[66.5-84.5] | 80.8[75.8-86.2] |
| 2011-2015 | 80.6[77.3-84.1] | 75.9[70.8-81.4] | 81.5[76.8-86.6] | 82.7[79.6-85.9] | 75.2[70.1-80.7]* | 75.3[67.9-83.6] | 78.8[71.8-86.4] | 84.9[80.8-89.2] |
| 2016-2020 | 85.7[83.1-88.5] | 81.0[77.2-85.1] | 84.6[80.3-89.1] | 85.7[83.2-88.3] | 84.6[80.8-88.6] | 85.0[79.8-90.7] | 80.6[75.0-86.5] | 87.3[83.8-90.8] |
| 5/1-year | | | | | | | | |
| 1971-1975 | 47.0 | 46.2 | 37.6 | 39.6 | 80.9 | 47.0 |  | 69.2 |
| 1976-1980 | 39.3 | 44.5 | 35.2 | 37.3 | 40.5 | 51.9 | 53.1 | 67.6 |
| 1981-1985 | 42.7 | 49.0 | 34.7 | 49.2 | 51.2 | 72.1 | 50.8 | 44.1 |
| 1986-1990 | 57.7 | 26.5 | 41.4 | 58.4 | 69.8 | 52.6 | 53.2 | 62.2 |
| 1991-1995 | 53.1 | 50.7 | 35.6 | 53.9 | 65.3 | 64.3 | 50.9 | 69.7 |
| 1996-2000 | 53.8 | 59.6 | 62.1 | 60.8 | 49.4 | 73.7 | 59.2 | 68.2 |
| 2001-2005 | 59.5 | 65.8 | 58.3 | 69.5 | 60.3 | 67.6 | 64.4 | 69.0 |
| 2006-2010 | 70.6 | 64.2 | 66.3 | 73.1 | 71.8 | 71.4 | 70.5 | 75.0 |
| 2011-2015 | 72.1 | 75.0 | 74.5 | 77.6 | 72.3 | 79.2 | 73.1 | 84.8 |
| 2016-2020 | 76.5 | 79.9 | 78.1 | 80.4 | 72.1 | 85.2 | 80.8 | 81.4 |
| Hypopharynx 1-year | | | | | | | | |
| 1971-1980 | 58.3[50.3-67.5] | 44.7[36.0-55.5] | 53.3[46.4-61.3] | 50.2[45.6-55.3] | 49.5[35.5-69.0] | 49.2[39.9-60.7] | 50.1[38.7-64.8] | 45.7[38.7-53.9] |
| 1981-1990 | 44.9[38.4-52.5] | 51.2[43.2-60.7] | 48.8[42.1-56.4] | 49.3[45.1-54.0] | 52.9[41.6-67.2] | 65.7[55.4-77.9] | 51.1[38.6-67.7] | 50.7[42.5-60.3] |
| 1991-2000 | 56.4[50.5-63.1] | 55.5[47.8-64.5] | 56.0[49.6-63.3] | 52.6[48.2-57.4] | 53.1[44.2-63.8] | 49.1[37.1-65.0] | 48.1[35.2-65.6] | 51.1[42.8-61.1] |
| 2001-2010 | 54.9[50.1-60.1] | 63.9[57.3-71.3] | 51.1[44.3-59.1]* | 53.5[49.0-58.5] | 55.0[46.2-65.5] | 59.9[46.6-76.9] | 52.8[41.0-67.9] | 59.0[51.1-68.1] |
| 2011-2020 | 61.5[57.8-65.4] | 58.9[53.1-65.3] | 66.7[59.9-74.2] | 58.0[52.9-63.7] | 66.5[59.5-74.3] | 70.3[57.6-86.0] | 51.5[38.8-68.4] | 61.5[53.3-71.1] |
| 5/1-year | | | | | | | | |
| 1971-1980 | 33.6 | 24.6 | 25.9 | 28.1 | 39.4 | 22.2 | 50.5 | 38.9 |
| 1981-1990 | 26.5 | 21.5 | 35.2 | 35.9 | 30.6 | 31.2 | 66.3 | 26.2 |
| 1991-2000 | 30.5 | 31.4 | 40.2 | 35.9 | 41.4 | 38.5 | 14.6 | 54.2 |
| 2001-2010 | 36.2 | 43.7 | 39.5 | 37.6 | 47.3 | 36.6 | 51.1 | 51.0 |
| 2011-2020 | 53.3 | 46.7 | 51.6 | 40.2 | 48.6 | 50.8 | 47.8 | 54.6 |
| Larynx 1-year | | | | | | | | |
| 1971-1975 | 88.5[85.1-92.1] | 83.8[77.7-90.3] | 86.9[82.6-91.4] | 87.2[83.9-90.5] | 79.2[70.5-89.0] | 62.3[43.0-90.1] | 84.7[72.1-99.4] | 84.7[74.4-96.5] |
| 1976-1980 | 83.9[80.9-87.0] | 82.0[77.2-87.0] | 90.1[86.5-93.8] | 88.4[85.6-91.2] | 84.7[77.3-92.7] |  |  | 87.2[79.0-96.2] |
| 1981-1985 | 83.3[80.2-86.4] | 85.9[81.9-90.1] | 85.4[81.3-89.7] | 87.0[84.4-89.8] | 82.5[75.6-90.0] | 80.6[69.7-93.2] | 82.5[73.0-93.2] | 91.3[85.3-97.8] |
| 1986-1990 | 83.3[80.2-86.5] | 81.2[77.1-85.6] | 84.8[81.1-88.7] | 87.4[84.7-90.0] | 82.9[76.9-89.4] | 73.1[60.8-88.0] | 84.3[71.9-98.9] | 88.2[82.0-94.9] |
| 1991-1995 | 84.2[81.3-87.2] | 86.7[83.0-90.6] | 84.5[80.6-88.5] | 87.3[84.6-90.1] | 78.7[72.1-85.8] |  | 85.2[77.2-94.1] | 90.7[84.8-97.1] |
| 1996-2000 | 82.4[79.6-85.4] | 84.8[80.8-89.0] | 83.2[79.5-87.0] | 88.1[85.5-90.8] | 73.7[66.9-81.1] |  | 80.5[72.8-88.9] | 87.2[81.1-93.7] |
| 2001-2005 | 85.2[82.3-88.3] | 81.9[77.7-86.3] | 87.8[84.5-91.2] | 85.7[82.9-88.5] | 82.5[76.9-88.5] |  | 86.0[78.2-94.6] | 82.6[76.4-89.2] |
| 2006-2010 | 84.9[82.3-87.6] | 83.3[79.6-87.2] | 87.4[83.9-91.2] | 87.9[85.3-90.7] | 81.0[75.3-87.0] | 87.0[78.0-96.9] | 85.4[77.6-93.9] | 81.9[75.7-88.5] |
| 2011-2015 | 87.3[84.7-89.9] | 84.0[80.0-88.2] | 89.4[86.4-92.4] | 89.7[87.3-92.2] | 84.3[78.6-90.4] |  | 74.8[65.2-85.9] | 88.8[82.8-95.2] |
| 2016-2020 | 87.7[85.4-90.0] | 83.5[80.0-87.1] | 89.0[85.9-92.2] | 89.9[87.5-92.3] | 87.1[81.2-93.4] | 87.1[80.4-94.4] | 83.4[76.0-91.6] | 83.8[76.9-91.4] |
| 5/1-year | | | | | | | | |
| 1971-1975 | 81.4 | 62.2 | 82.0 | 77.8 | 71.6 | 84.3 | 77.1 | 87.5 |
| 1976-1980 | 66.4 | 62.2 | 79.7 | 72.1 | 74.6 |  |  | 78.9 |
| 1981-1985 | 70.8 | 66.8 | 66.3 | 76.8 | 76.0 | 75.8 | 71.6 | 63.7 |
| 1986-1990 | 69.0 | 71.3 | 78.9 | 78.3 | 62.6 | 60.9 | 61.4 | 81.1 |
| 1991-1995 | 67.2 | 69.4 | 74.7 | 78.0 | 69.3 |  | 66.4 | 77.6 |
| 1996-2000 | 75.0 | 75.1 | 76.8 | 77.5 | 67.8 |  | 77.1 | 79.7 |
| 2001-2005 | 67.4 | 75.1 | 76.8 | 77.0 | 73.7 |  | 80.5 | 66.3 |
| 2006-2010 | 76.0 | 70.3 | 78.1 | 79.3 | 72.1 | 77.9 | 70.0 | 78.9 |
| 2011-2015 | 75.4 | 70.5 | 77.2 | 78.5 | 70.1 |  | 71.7 | 74.3 |
| 2016-2020 | 79.1 | 73.3 | 83.4 | 78.8 | 79.7 |  | 84.9 | 66.0 |


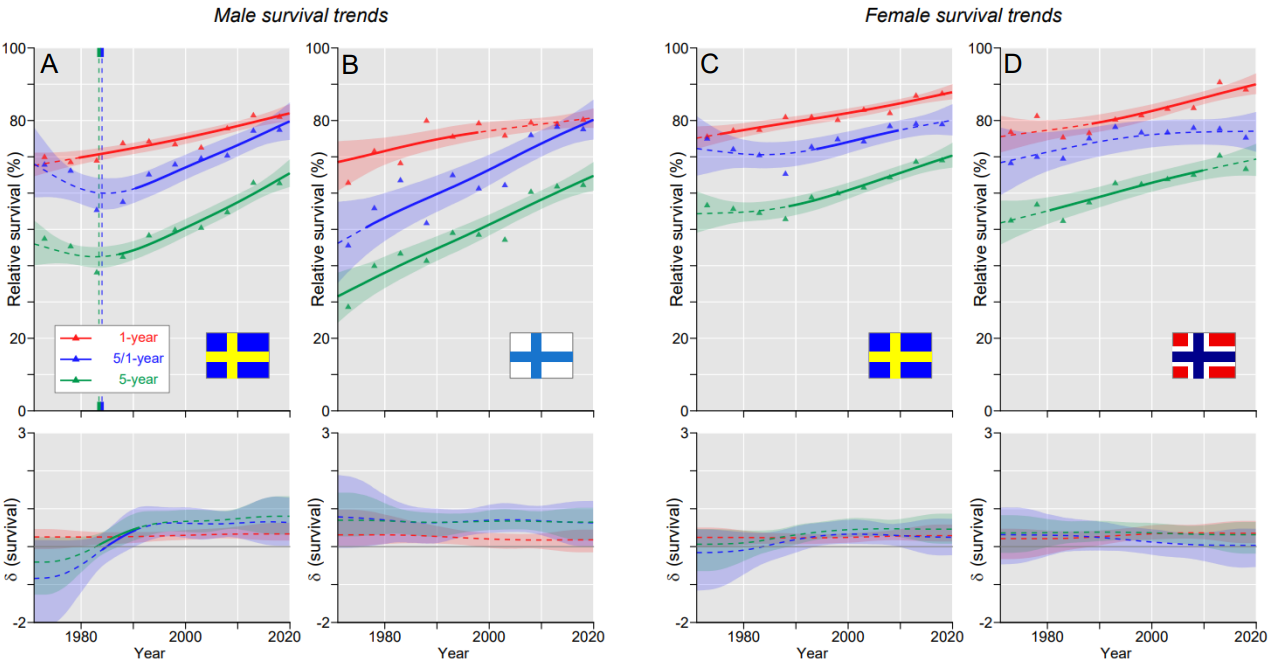


SuppFig.1. Oral cancer: relative 1- , 5/1- and 5-year survival in Swedish (A) and Finnish men (B), and in Swedish (C) and Norwegian women (D).


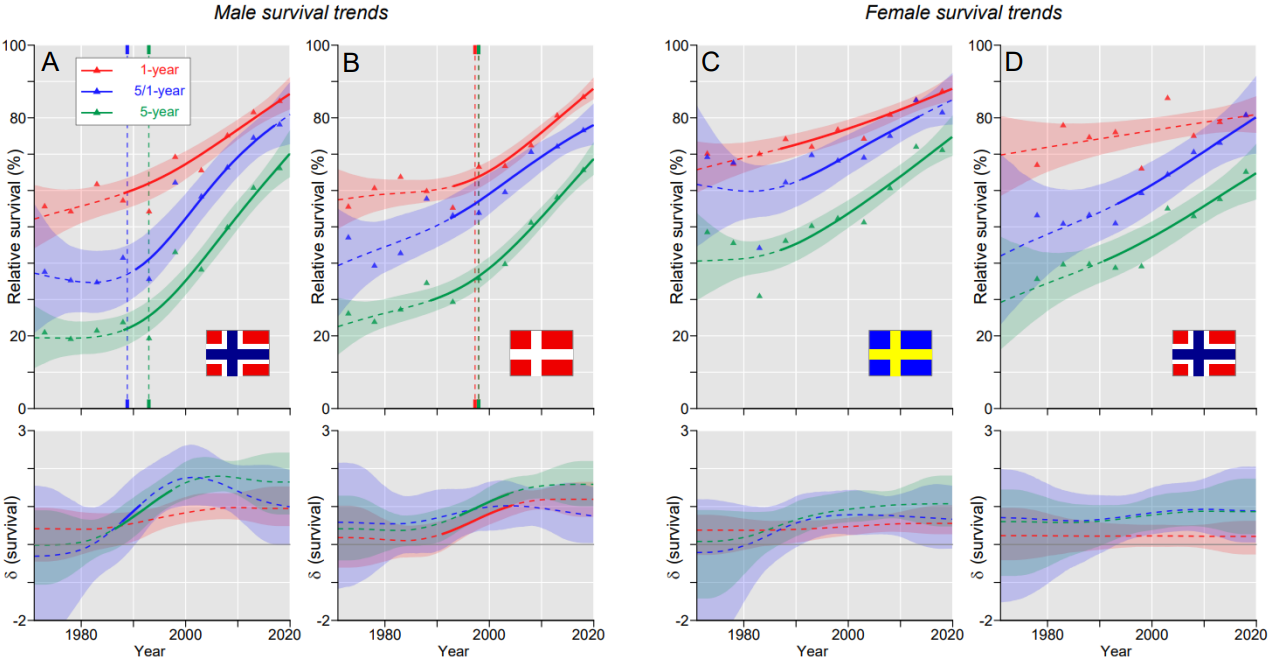


SuppFig.2. Oropharyngeal cancer: relative 1- , 5/1- and 5-year survival in Norwegian (A) and Danish men (B), and in Swedish (C) and Norwegian women (D).


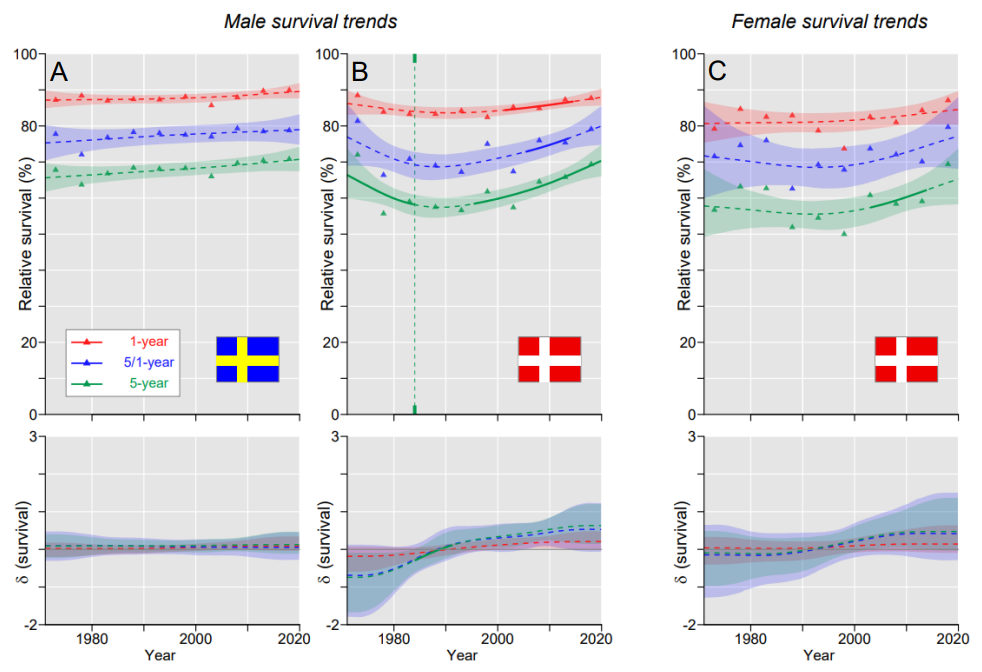


SuppFig.3. Laryngeal cancer: relative 1- , 5/1- and 5-year survival in Swedish (A) and Danish men (B), and in Danish women (C).
